# Thin-Film Nanocomposite (TFN) Membranes for Water Treatment Applications: Characterization and Performance

**DOI:** 10.3390/membranes13050477

**Published:** 2023-04-28

**Authors:** Amr Tayel, Ahmed B. Abdelaal, Amal M. K. Esawi, Adham R. Ramadan

**Affiliations:** 1 Department of Chemistry, The American University in Cairo, AUC Avenue, New Cairo 11835, Egypt; 2 Department of Chemistry, McGill University, 845 Rue Sherbrooke O, Montreal, QC H3A 0G4, Canada; ahmed.bahaa-eddin@mail.mcgill.ca; 3 Department of Mechanical Engineering, The American University in Cairo, AUC Avenue, New Cairo 11835, Egypt; a_esawi@aucegypt.edu

**Keywords:** thin-film nanocomposite membranes, nanofillers, water treatment, membrane characterization, membrane performance

## Abstract

Thin-film nanocomposite (TFN) membranes have been widely investigated for water treatment applications due to their promising performance in terms of flux, salt rejection, and their antifouling properties. This review article provides an overview of the TFN membrane characterization and performance. It presents different characterization techniques that have been used to analyze these membranes and the nanofillers within them. The techniques comprise structural and elemental analysis, surface and morphology analysis, compositional analysis, and mechanical properties. Additionally, the fundamentals of membrane preparation are also presented, together with a classification of nanofillers that have been used so far. The potential of TFN membranes to address water scarcity and pollution challenges is significant. This review also lists examples of effective TFN membrane applications for water treatment. These include enhanced flux, enhanced salt rejection, antifouling, chlorine resistance, antimicrobial properties, thermal stability, and dye removal. The article concludes with a synopsis of the current status of TFN membranes and future perspectives.

## 1. Introduction

Access to freshwater is currently among the most significant environmental challenges facing the global community. The scarcity of freshwater in some areas is partly due to the rapid expansion of the agricultural and industrial sectors, which has led to an increased demand for desalination. Water treatment can be broadly categorized into three classes: chemical, physical, and biological treatments [1]. Among these classes, membrane-based separation technologies, such as reverse osmosis (RO) and nanofiltration (NF), are playing increasing roles in the production, reuse, and desalination of water [2]. The RO and NF membranes used for seawater desalination and water reuse typically possess a thin-film composite (TFC) structure that is composed of a porous substrate and an ultrathin polyamide (PA) selective layer, which is approximately 10–400 nm thick [3].

**Figure 1:**
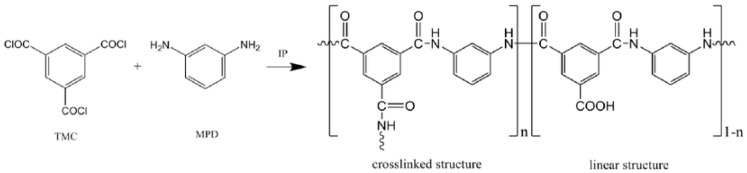
Diagram depicting a typical IP reaction [6].

Since the development of thin-film composite (TFC) membranes in 1970 by Cadotte, these membranes have frequently been used for industrial and research applications [4]. TFC membranes benefit from a PA layer atop a support layer, providing improved salt rejection and separation performance. The formation of this layer is typically accomplished using an interfacial polymerization (IP) reaction atop the support, by reacting an acyl chloride moiety with an amine moiety to form a cross-linked PA layer. Trimesoyl chloride (TMC) is primarily used as the triacyl chloride group in the reaction, and piperazine (PIP) or m-phenylenediamine (MPD) are generally used as the amine groups in IP reactions; however, various amine-based monomers have been explored and reported in the literature. For example, Gallardo et al. formulated a PA layer based on triethylenetetramine (TETA) and TMC using a vacuum-assisted IP approach [5]. Figure 1 below illustrates a typical IP reaction [6].

In addition to the choice of monomer for constructing the PA layer, it is crucial to note that the cross-linking structure of the PA layer can have a marked effect on the morphology and behavior of a TFC membrane. Numerous studies have investigated the relationship between the cross-linking degree of the IP layer and the performance of the TFC membranes [7,8]. The majority of commercial RO membranes are categorized as TFCs [9].

The acyl chloride group (TMC) is typically prepared in an organic solution, while the diamine molecule (MPD) is generally dissolved in an aqueous solution.

There are numerous variables that can influence the desalination characteristics and morphology of the PA layer, such as monomer concentrations, curing temperature, and curing time. A study conducted by Wei et al. measured the morphological changes of a TFC membrane upon altering the monomer concentration of TMC and MPD [10]. The authors concluded that the ratio of MPD/TMC concentrations correlated with the degree of cross-linking and that increasing the ratio would be met with an increase in the salt rejection. The authors additionally concluded that a lower MPD/TMC ratio yielded unreacted acyl chloride functionalities that led to less cross-linking. Equipped with AFM measurements and SEM imaging, Zhan et al. measured the changes in surface roughness of a polypiperazine PA layer atop a polyethersulfone (PES)-based support as a function of the curing temperature and curing time [11]. The authors noted that the water flux appeared to decrease at higher curing temperatures, and that the surface roughness increased at curing times of 1–5 min.

In current TFC research, the literature indicates various innovative methods for the formation of the PA layer, ranging from the use of sacrificial layers to the incorporation of new surface-coating techniques for PA layer synthesis. In addition to these classes of TFC membranes, other innovations include embedding nanomaterials within the PA layer (discussed in detail throughout this review), as well as manipulating the conditions of the IP reaction [4]. In an example of the latter approach, Tan et al. reported the tuning of an IP reaction by modifying the reaction conditions to synthesize patterned PA layers atop a polysulfone (PSf) support [12]. The authors recognized that the interfacial polymerization process was largely facilitated in the organic component of the reaction. Hence, by adding polyvinyl alcohol (PVA), the diffusion characteristics of the amine moiety were minimized, leading to a “diffusion-driven instability”. By exerting control on the diffusion of the amine monomer, the authors produced PA surfaces with various patterned structures that were imaged using transmission and scanning electron microscopy (TEM and SEM, respectively).

In an example of a surface-coating technique used for PA layer formation, Joung-Eun et al. employed a molecular layer-by-layer (mLbL) technique atop a polyacrylonitrile (PAN) membrane support [13]. Using mLbL deposition, the authors reported an increased smoothness and decreased thickness of their PA layer, accompanied by more favorable sodium chloride (NaCl) rejection characteristics. In another such example of surface-coating methodologies, Wang et al. used an atomic-layer deposition method to synthesize a polyimide layer atop porous alumina for filtration applications [14]. Using this method, the authors noted a bovine serum albumin (BSA) protein retention of 82% with a permeation rate of 800 LMHbar^−1^.

Using nanoimprint lithography (NIL), Maruf et al. facilitated an IP reaction on nanoimprinted PES ultrafiltration (UF) support membranes for RO desalination. By tuning the IP reaction parameters as well as the topographical features of the support membrane, the authors concluded that the topography of the patterned PA layer could be precisely tuned for a variety of applications [15]. In an example of sacrificial layer formation, Karan et al. formed a cadmium hydroxide layer on an alumina support for filtration applications [16]. Through the formation of this sacrificial layer, the authors produced a 10 nm-thick PA layer with a stark improvement in the acetonitrile permeance (112 LMHbar-1) compared to commercial TFC membranes.

Polyamide-based TFC membranes have limitations in practical applications due to their poor chlorine tolerance, low chemical stability, and high fouling tendency. Their highly cross-linked and amorphous structure also lacks porosity and interconnectivity, resulting in a trade-off between water permeability and membrane selectivity. High salt rejection often implies a low water permeability [17,18]. While there are numerous examples of innovative strategies for PA formation, the research in the area of thin-film nanocomposite (TFN) membranes is vast, and demonstrates the versatility and performance advantages of nanomaterial incorporation within membranes [19]. The concept of a TFN membrane was first introduced by Hoek in 2007, and has since been extensively studied for preparing reverse osmosis and nanofiltration membranes [20]. In the IP process, nanomaterials are incorporated into the dense PA layer to enhance membrane properties, including hydrophilicity, chlorine resistance, and anti-fouling properties. The size, chemical structure, and loading amount of nanoparticles in the PA layer affect the TFN membrane’s PA network structure, surface hydrophilicity, thickness, permeability, and rejection properties [21].

Reviews on TFN membranes have recently been published by Bhaskar et al. [21], Kumar et al. [22], Liao et al. [23], Kadhom et al. [24], Krishnan et al. [25], Zhao et al. [26], and Li et al. [27]. Comprehensive reviews were presented on the use of nanofillers as quantum dots [28], metal–organic frameworks (MOFs) [29], organic-based nanomaterials [30], nanomaterial positions and dimensions [31], and on TFN membranes with an interlayered structure [32,33], for RO desalination [34], and for pervaporation applications [35], loose nanofiltration [36], thermal stability [37], and surface characterization using contact angle techniques [38]. Additionally, numerous advancements have been made in the methodology of embedding nanofillers in TFNs. These include pressure-assisted polydopamine (PDA) modification for improved hydrophilicity and anti-fouling, as well as the incorporation of tannic-acid-functionalized MOFs for optimized water permeance and salt rejection [39,40]. With the significant increase in the number of publications on TFN membranes, this updated review is considered timely and of wide interest.

This review aims to update researchers in the field of TFN membranes with the latest developments and provide newcomers with an up-to-date summary of TFN membrane synthesis and applications. A unique feature of this review is a detailed characterization section that provides guidance on commonly used techniques for analyzing the structural, compositional, mechanical, and surface properties of TFN membranes. Furthermore, the review summarizes recent applications of TFN membranes in water treatment, with a focus on emerging applications.

## 2. Thin-Film Nanocomposite Membranes

The following sections outline representative examples of nanofillers used in the fabrication of TFN membranes. Although the scope of this review includes a broad range of nanofillers, the rapid progress in this area and the ongoing integration of newly developed nanofillers pose a challenge in achieving a comprehensive inclusion of all used nanofillers. A summary of the performance of TFN membranes based on the type of the used nanofiller is given in Table 1.

### 2.1. Incorporation of Inorganic Nanofiller

Yang et al. incorporated silver nanoparticles (Ag NPs) in TFN membranes in order to improve the anti-fouling properties of TFC membranes. The addition of Ag NPs in the polyamide (PA) layer formed nanochannels that tripled the water permeance and increased the rejection of NaCl, boron, and small molecular organic compounds. However, the antifouling properties of the membranes were not fully characterized in this work [41].

Titanium oxide (TiO_2_) is used for membrane modification due to its anti-biofouling properties. TiO_2_ NPs release oxidative species that can destroy micro-organisms and prevent biofilm formation. TiO_2_ NPs were incorporated into PA layers to enhance the performance of thin-film nanocomposite (TFN) membranes in water treatment applications. The integration of TiO_2_ NPs into the PA layer was investigated by dispersing TiO_2_ NPs in either the organic phase of TMC or the aqueous phase of MPD before undergoing the interfacial polymerization process. The TFN membranes with TiO_2_ NPs had improved water permeability, flux, and salt rejection compared to neat TFC membranes [42]. The addition of TiO_2_ NPs also increased the thermal stability and anti-biofouling properties of the TFN membranes as demonstrated by Khorshidi et al. [43].

Wang et al. investigated the use of cerium oxide (CeO_2_) NPs as potential nanofillers in PA layers for RO membranes. Novel TFN membranes were created by incorporating CeO_2_ NPs into the ultra-thin PA layers, resulting in increased hydrophilicity and water permeance without negatively affecting membrane selectivity. The presence of CeO_2_ NPs also mitigated organic fouling in the membranes [44].

In a recent study by Al-Gamal et al., modified silica (m-silica) was functionalized using (3-amino- propyl) triethoxysilane (APTES), aspartic acid (AA), and hexamethylenediamine (HMDA), then embedded into the PA layer to improve the membrane performance. The m-silica-incorporated membranes exhibited improved hydrophilicity, porosity, roughness, surface charge, and water permeability compared to the bare membranes [45]. Another study by Wu et al. involved incorporating alkyl-capped silica NPs into the organic phase during interfacial polymerization to tailor the microstructures of the PA layer in TFN membranes. The alkyl group modification also reduced nanoparticle agglomeration and enhanced compatibility with the PA matrix. This modification improved the surface charge and surface area, resulting in increased water flux and high salt rejection rates for both brackish and seawater desalination applications [46].

**Table 1:** Summary of performance of TFN membranes (based on the nanofiller type). (A) indicates the dispersion of the nanofiller in aqueous phase, and (O) indicates the dispersion of the nanofiller in the organic phase.

Nanofiller	Average Size (nm)	Loading at Best Performance	Feed Solution Conc.	Pressure (Bar)	Membrane Type	PWP LMH/Bar (From → to) Best Performance	% Salt Rejection (From → to) Best Performance	Reference	Notes
Inorganic Nanofillers									
TiO_2_ nanoparticles (NPs)	10	0.0125 wt/v% (O)	2000 ppm NaCl	15.2	RO	1.41 → 1.60	97.9 → 97.7	[43]	Thermal stability and anti-biofouling
Fluorinated SiO_2_ NPs	150–200	0.12 wt/v% (O)	2000 ppm NaCl	15.5	RO	2.66 → 2.50	96.0 → 98.6	[47]	Hydrophobic NPs to improve salt rejection
CeO_2_ NPs	54	0.01 wt/v% 100 mg/L (A)	2000 ppm NaCl	16.0	RO	1.84 → 2.75	98.7 → 98.0	[44]	Rougher surfaces and thinner PA layers—antifouling
CeO_2_ NPs	--	0.2 wt/v% (O)	2000 ppm NaCl	20.0	NF	1.66 → 2.06	~ 85.5 → 94.8	[48]	Antifouling
FeO	50	0.2 wt/v% (O)	2000 ppm NaCl	10.3	NF	1.7 → 2.3	66.2 → 92.1	[49]	Antifouling
Ag NPs	--	0.2 wt/v% Preloaded on PSf	2000 ppm NaCl	20.0	RO	0.93 → 2.50	97.4 → 99.1	[41]	Formation of nanochannels around the Ag NPs
Halloysite nanotubes (HNT)-COOH	28 (inner diameter)	0.05 wt/v% (O)	3000 ppm NaCl	20	RO	1.31 → 2.48	99.1 → 99.1	[50]	Leaching test
Cu NPs nanovoids	10–21	Preloaded on PSf	2000 ppm NaCl	20	RO	0.76 → 1.26	96.7 → 95	[51]	Removed by acid etching
MgFe_2_O_4_ NPs	21	0.005 wt/v% (A)	2000 ppm NaCl	10	NF	3.71 → 5.05	51.0 → 69.1	[52]	Antibacterial
Si NPs	50	0.07 wt/v% (O)	1000 ppm Na_2_SO_4_	6	NF	6.98 → 9.85	98.5 → 98.3	[53]	Antifouling BSA
Si NPs	--	0.02% *w/v* (O)	2000 ppm NaCl	5–30	RO	4.80 → 8.20	98.0 → 98.0	[54]	In-situ prep.
Aminophenyl-modified MSN (AMSN)	40	0.025% *w/v* (A)	32,000 ppm NaCl	55.2	RO	0.83 → 1.00	99.3 → 99.0	[55]	Lower flux than expected
Aminated TiO_2_ NPs	123	0.3% *w/v* (A)	1000 ppm Na_2_SO_4_, NaCl	5	NF	3.90 → 10.40	32.1 → 18.6 (NaCl) 97.8 → 98.0 (Na_2_SO_4_)	[56]	Monovalent/Divalent salt separation
TiO_2_ NPs	<100 nm	0.1% *w/v* (O)	2000 ppm NaCl	20	RO	1.93 → 3.14	97.2 → 97.0	[57]	Antifouling (humic acid); antibacterial
ZnO NPs	15–20	0.02% *w/v* (A)	2000 ppm NaCl	20	RO	0.72 → 1.19	99.2 → 97.0	[58]	Antifouling (humic acid); antibacterial
Alkyl-silica NPs	20	0.5 wt/v% (O)	2000 ppm NaCl	15.5	RO	3.4 → 3.57	99.5 → 99.6	[46]	Boron removal
			32,000 ppm NaCl	55.0			98.6 → 99.4		
Amine-rich synthetic talc (NHST) nanosheets	---	0.5 wt/v% (A)	2000 ppm Na_2_SO_4_	5	NF	16.0 → 24.45	87.00 → 98.96	[59]	Antifouling (BSA); antibacterial
TiO_2_	5–10	0.015 wt/v% (A)	1000 ppm Na_2_SO_4_	5	NF	12.5 → 20.3	95.5 → 91.1	[60]	Electrospray
Hierarchical nanosized zeolite	200–800	0.005 wt/v% preloaded on PSf	1000 ppm Na_2_SO_4_	4	NF	13.1 → 23.2	98.5 → 97.5	[61]	* Vacuum filtration * Stability
Flower-like MnO_2_ NPs	200–500	0.5 wt/v% (A)	20 ppm Rhodamine B dye	10	NF	7.72 → 11.88 Methanol permeability	99.2 → 96.94	[62]	OSN
Modified silica (m-silica) NPs	---	0.4 wt/v% (A)	1000 ppm Na_2_SO_4_	10	NF	3.63 → 6.16	23.32 → 97.96	[45]	Stability
Carbon-based Nanofillers									
N-GO quantum dots (QD)	3–8	0.02 wt/v% (A)	2000 ppm NaCl	15.0	RO	0.62 → 1.66	~ 93.0 → 93.0	[63]	Improved thermal stability
Na-C QDs	2–6	1 wt/v% (A)	2000 ppm NaCl	15.0	RO	1.74 → 2.56	97.7 → 97.7	[64]	Stable at 23 bar
Cellulose nanocrystals (CNCs)	15	0.1 wt/v% (O)	3000 ppm NaCl	20	RO	1.50 → 3.15	98.5 → 97.8	[65]	
Graphene oxide (GO)	--	0.00087 wt/v% Preloaded on PES	1000 ppm Na_2_SO_4_	8.0	NF	3.11 → 4.04	94 → 95.8	[66]	Vacuum filtration; antifouling test (dyes)
GO	--	0.0013 *w/v* % Preloaded on PES	1000 ppm Na_2_SO_4_	8.0	NF	1.80 → 4.13	95.0 → 96.0	[67]	Vacuum filtration; antifouling BSA, RB5
Hollow porous carbon spheres (HPCSs)	150	0.02 wt/v% (A)	20 ppm Rhodamine B dye	10	NF	8.09 → 11.51 Methanol permeability	99.0 → 97.50	[68]	OSN
Acrylic-acid-coated GO	---	0.0013 wt/v% Preloaded on PSf	1000 ppm Na_2_SO_4_	8	NF	9.15 → 8.41	97.00 → 98.69	[69]	* Prepared at 55 °C * Antifouling BSA * Textile saline
Liposomes	130–160	0.02 wt/v% (A)	1000 ppm MgCl2	2	NF	11.17 → 18.21	90.1 → 95.9	[19]	Ion selectivity
Nanodiamond (ND)	50	0.02 wt/v% (A)	1420 ppm Na_2_SO_4_ (10 mM)	6	NF	4.7 → 15.0	98.0 → 97.3	[3]	
Carboxylated cellulose nanocrystals (C–CNCs)	100–500	0.05 wt/v% (A)	2000 ppm Na_2_SO_4_	6	NF	8.2 → 10.4	95.0 → 98.3	[70]	
Carboxylated multiwalled carbon nanotubes (MWCNTs)	50–200	0.01 wt/v% (A)	1169 ppm NaCl (20 mM)	2.5	NF			[71]	FO
Hybrid Nanofillers									
Metal organic frameworks (MOFs)-UiO-66	145	0.005 wt/v% (O)	1000 ppm NaCl	2	FO	1.87 → 4.47	96.3 → 96.7		
MOF/UiO-66	50	0.05 wt/v% (O)	2000 ppm NaCl	15.5	RO	2.37 → 3.67	99.1 → 99.4	[72]	Boron removal
			32,000 ppm NaCl	55.0	RO	51.46 → 61.32	99.05 → 99.27		Doping in hexane before TMC
MOF/UiO-66-NH_2_	200	0.01 wt/v% (A)	1500 ppm Na_2_SO_4_	4	NF	14.50 → 30.80	99.0 → 97.5	[73]	Vacuum filtration
MOF/UiO-66-NH_2_	100	0.02 wt/v% (A)	100-ppm malachite green dye	10	NF	1.11 → 1.33	85.3 → 91.9	[74]	Antifouling (humic acid); antibacterial
MOF/ZIF-8	150	0.2 wt/v% (A)	2000 ppm NaCl	15.5	RO	2.76 → 3.95	98.9 → 99.2	[75]	Investigated dispersion of ZIF-8 in organic and aq. solvents
MOF/ZIF-8	60	0.05 wt/v% Preloaded on PSf	2000 ppm NaCl	15.5	RO	2.86 → 3.72	96.6 → 97.8	[76]	Spray coating
MOF/ZIF-8	70	Preloaded by dip coating	20 ppm Sunset Yellow/Methanol	20	NF	5.8 → 8.7	96.5 → 90.0	[77]	
MOF/ZIF-67	240	Preloaded by dip coating	20 ppm Sunset Yellow/Methanol	20	NF	5.8 → 4.8	96.5 → 79.3	[77]	
MOF/ZIF-93	67	0.2% wt/v% (O)	1 ppm Diclofenac	20	NF	6.8 → 24.2	99.3 → 99	[78]	Interfacial synthesis
MOF/HKUST	800	0.2% wt/v% (O)	1 ppm Diclofenac	20	NF	6.8 → 33.1	99.3 → 99.5	[78]	Interfacial synthesis
(Polydopamine) PD/MOF/ZIF-8	150	0.005 wt/v% Preloaded on PSf	1000 ppm Na_2_SO_4_	4	NF	33.8 → 53.5	99.2 → 95.3	[79]	Also used ZIF-67 and CaCO3 as sacrificial template
Covalent organic framework nanosheets (CONs)	250 nm (lateral size)	0.01 wt/v% Preloaded on PSf	1000 ppm Na_2_SO_4_	2	NF	15.7 → 53.6	85 → 94.3	[80]	Vacuum filtration; thinner skin layer to sub 10 nm
Camphor- Al_2_O_3_ NPs	11–20	0.5 wt/v% (O)	2000 ppm Na_2_SO_4_, NaCl	10.0	NF	3.02 → 7.88	96.5%, 92.4%	[81]	Long term stability of membrane for 10.5 h
PMSA * -g-GO (Zwitterion-GO)	--	0.02 wt% (A)	1000 ppm NaCl	12	RO	0.85 → 1.47	93.8 → 94.8	[82]	Antifouling
Ag/carbon nanotube (CNT)/PDA	--	Preloaded on PES	2000 ppm Na_2_SO_4_,	10.0	NF	0.54 → 1.16	99.4 → 94.1	[83]	Vacuum filtration
Al_2_O_3_/CNT/PDA	--	Preloaded on PES	2000 ppm Na_2_SO_4_,	10.0	NF	0.54 → 1.17	99.4 → 95.0		
Fe_2_O_3_/CNT/PDA	--	Preloaded on PES	2000 ppm Na_2_SO_4_,	10.0	NF	0.54 → 1.05	99.4 → 96.7		
TiO_2_/CNT/PDA	--	Preloaded on PES	2000 ppm Na_2_SO_4_,	10.0	NF	0.54 → 0.96	99.4 → 96.9		
Single-wall carbon nanotube (SWCNT)/PDA	CNT diameter, <2 nm effective surface area: 12.56 cm^2^	0.01 wt% Preloaded on PES Vacuum filtration	2000 ppm Na_2_SO_4_ 100 ppm Methyl orange (MO) and other dyes	5.0	NF	6.5 → 21	R_MO_, 82 → 91.5 R_Na2SO4_, 98.5	[84]	Selectivity of Cl^−^/SO_4_^2−^ is ~85.5 Selectivity of NaCl/MV is >123.5
Ag-ZnO	37	1.00 wt/v% (A)	5.0 ppm 2,4-DCP	11.0	NF	0.90 → 1.90	58.0 → 85.0	[85]	Antifouling silver leaching studies
MOF/MIL-53 (Al)	12	0.20 wt/v% (O)	584.4 ppm NaCl	5.0	FO	0.39 → 1.74	80.3 → 92.1	[86]	Doxycycline removal
Cu-Al layered double hydroxide (LDH) nanofillers	--	0.10 wt/v% (O)	1000 ppm Na_2_SO_4_	7.0	NF	3.18 → 7.01	98.0 → 98.0	[87]	Antifouling CTAB
PDA-SiNPs	60	0.07 wt/v% (O)	1000 ppm Na_2_SO_4_	6	NF	6.98 → 20.00	95.0 → 94.5	[88]	Antifouling BSA
Ni-MOFs	--	0.015% *w/v* (A)	4000 ppm NaCl	20	RO	1.03 → 2.52	99.3 → 99.2	[89]	Antifouling (humic acid)
Ag-MOFs	439	0.01% *w/v* (A)	10 ppm Na_2_SO_4_	2	NF	9.2 → 14.3	54.0 → 84.1	[90]	Antibacterial
			10 ppm RB dye			9.2 → 14.3	92.8 → 99.3		
ZIF-8	100	0.05 wt/v% Preloaded on PSf	2000 ppm NaCl	15.5	RO	0.95 → 3.74	98.47 → 99.03	[17]	
GO@CS	200	0.01 wt/v% (A)	2000 ppm NaCl	20	RO	0.73 → 1.58	98.35 → 99.10	[91]	Stability
Ag@ZnO-OAc	8	0.3 wt/v% (O)	1000 ppm Na_2_SO_4_	5	NF	7.2 → 5.5	94.5 → 98.8	[92]	Antifouling BSA
Palygorskite/Ag clay nanotubes	30–60	0.00075 wt/v% (A)	2000 ppm NaCl	16	RO	1.5 → 2.5	98.6 → 98.3	[93]	Antibacterial * Stability

* PMSA stands for poly(2-(Methacryloyloxy)ethyl dimethyl-(3-sulfopropyl)ammonium hydroxide).

### 2.2. Incorporation of Carbon-Based Nanofillers

Carbon nanotubes (CNTs) have a strong potential for use in TFN membranes due to the similarity of their tubular structure to the water transport channels in biological membranes. However, achieving an ordered alignment of CNTs within the PA matrix has been a challenge [94]. Zhao et al. have successfully prepared hydrophilic CNTs using surface modification and embedded them into the PA layer during interfacial polymerization, resulting in TFN membranes with higher water permeance and maintained selectivity. Carboxyl-functionalized multi-walled carbon nanotubes (MWCNTs) introduced into the PA structure also resulted in TFN membranes with improved antifouling properties and chlorine resistance. The improved antifouling properties are attributed to greater surface hydrophilicity and more negative surface charges upon the introduction of MWCNTs [95]. Carboxylated-MWCNTs nanofillers were investigated for forward osmosis (FO) applications by Rashed et al. The results showed that the incorporation of carboxylated MWCNTs in the PA layer improved the pure water flux and reduced the reverse solute flux for the prepared TFN membranes [71].

The use of graphene oxide (GO) as nanofillers has shown improvements in the performance of various TFN membranes. GO has an abundance of surface functional groups, a high specific surface area, and exceptional mechanical and thermal properties. GO can enhance water transport by creating interlayer channels, allowing for fast diffusion. In addition, GO nanosheets are considered non-depleting nanomaterials, compared to silver nanoparticles, that inactivate bacteria through direct contact. In an investigation by Lai et al., GO was embedded in the PA layer, resulting in improved water permeability and NaCl rejection [66,67].

Carbon quantum dots (CQDs) and cellulose nanocrystals (CNCs) are carbon nanomaterials with desirable properties such as small size and good biocompatibility. They can be modified with functional groups, such as amine groups, to enhance their stability and performance in TFN membranes [63]. Gai et al. functionalized CQDs with Na^+^ and introduced them into the PA layer, which significantly increased pure water permeance while maintaining salt rejection [64]. Asempour et al. mixed CNCs into the PA active layers, resulting in a doubled permeate flux with a slight decrease in salt rejection. Both approaches improved the performance of TFN membranes for desalination and filtration applications [65].

### 2.3. Incorporation of Hybrid Nanofillers

Metal–organic frameworks (MOFs) are hybrid materials composed of inorganic metal centers connected by organic linkers that form flexible frameworks with porous structures. Due to their large surface area, porosity, variable pore size, and adjustable surface functionality, MOFs are promising porous-fillers in TFN membranes. Zeolitic imidazolate frameworks (ZIFs) are a type of MOFs that consist of tetrahedral metal ions and imidazole-based organic linkers. ZIFs have enduring porosity, high mechanical strength, and improved chemical stability [96,97].

Lee et al. and Hu et al. studied the effect of ZIF-8 particle size on the performance of TFN membranes. They found that the size of ZIF-8 affected surface coverage and external surface area per particle. The TFN membrane incorporated with ZIF-8 with an average size of 150 nm had the highest water permeance and NaCl rejection compared to other sizes [17,75]. Liu et al. investigated the use of UiO-66 as a nanofiller in TFN membranes for nanofiltration, forward osmosis, and RO. They found that the UiO-66-blended TFN membrane showed an increase in water flux and marginal increase in rejection compared to a benchmark TFC membrane. The modified membrane also exhibited enhanced boron rejection due to the chemisorption of UiO-66 with boric acid [72]. Comparable results were reported by Gohain et al. [74].

In their investigation, Huang et al. studied the incorporation of hydrophobic Ag@ZnO-Oleic acid (OAc) nanoparticles into the PA layer of TFN membranes. The resultant TFN membranes exhibited excellent antibacterial performance as well as improved salt rejection and antifouling properties compared to the bare TFC membranes [92]. Other hybrid nanofillers were studied to improve TFN membrane performance such as camphor-Al_2_O_3_NPs [81], 2D-MOF nanosheets [89], clay/silver NPs [93], and GO@chitosan core-shell nanocomposites [91].

## 3. Membrane Characterization

The properties of thin-film composite (TFC) and thin-film nanocomposite (TFN) membranes, as well as their performance, generally depend on their morphology, structure, and surface properties. Therefore, different techniques are utilized in membrane characterization. Below is a summary of the commonly used techniques together with representative examples of their use. The common thread for membrane characterization is the comparison of the TFN membranes to the corresponding bare TFC ones, thus giving useful insights into the changes occurring in the PA layer with the addition of the nanomaterials.

### 3.1. Structural and Elemental Analysis

#### 3.1.1. X-ray Diffraction (XRD)

XRD provides useful information about the phase structure and purity of crystalline materials. [98]. In their investigation, Asempour et al. compared the XRD spectra of the bare TFC and the 0.1%-CNC-TFN membranes. The presence of the additional XRD peaks in the TFN membrane at 14° and 16.5° confirmed the successful incorporation of CNCs nanofillers into the polyamide (PA) layer [65]. Other peaks at 18.5°, 21.5°, and 23.8° are consistent with the XRD spectrum of the α-crystalline phase of the PSf support [59,65].

Dai et al. reported that the incorporation of MOF nanofillers in the PA layer showed an intense peak at 14.2° compared to the bare TFC membrane. The authors argued that this XRD peak indicated a parallel configuration of the nanofiller to the membrane surface with additional pores that improved the water permeability through the TFN membrane [99]. The successful embedding of ZnO nanofillers in the PA layer is confirmed by an additional XRD peak at 36.4° which is absent in the TFC membrane XRD spectrum. This peak is attributed to the (011) plane of the ZnO nanocrystals [58]. However, due to the low amount of nanofiller used, XRD was reported in other studies to fail to detect the presence of the nanofiller in the PA layer [77,100].

Small-angle X-ray scattering spectrometer (SAXS) was employed by Wu et al. to analyze the structure variation in the PA layer after adding alkyl-silica nanofillers [46]. By obtaining various useful parameters using SAXS, such as the gyration radius, primary structure radius, and chain distance, the authors demonstrated the successful incorporation of the alkyl-silica nanofillers in the polymer network. This was justified by the gradual increase in the gyration radius with increasing amounts of nanofiller, combined with the relatively unchanged values of the primary structure radius and chain distance for each sample.

#### 3.1.2. X-ray Photoelectron Spectrometer (XPS)

XPS is used for the elemental analysis of different nanocomposite materials. In the context of TFN analysis, it provides useful information about the surface elemental composition of the TFC/TFN membrane [101,102]. Detailed analysis of the XPS spectra of TFC membranes can be found elsewhere [59,62,68,74]. Typically, three significant peaks are present for TFC/TFN membranes at 284.6, 400.5, and 532.0 eV, which correspond to the C, N, and O elements, respectively [61,103].

The increase in the O/N and C/N ratio of the TFN membrane is associated with decreased degrees of cross-linking and enhanced water permeability during PA layer formation [103,104]. Wang et al. attributed this phenomenon to the decreased diffusion of the MPD amine monomer towards the organic component due to NP agglomeration [44]. Similar results were reported by Wang et al. [61] and Huang et al. [92]. The O 1s XPS spectrum of the TFC membranes confirmed the formation of the amide bond during the IP process. The O 1s XPS spectrum showed two peaks at 531.2 eV and 532.2 eV. The former peak was ascribed to the C-O, C=O, and O=C-N bonds and the latter peak originated from H…O=C-N and O=C-O bonds. The incorporation of NPs in the TFN shifted both peaks to a higher binding energy and introduced an additional peak at 530.7 eV which is consistent with the successful incorporation of CeO_2_ NPs in the PA layer [44].

The increased amount of oxygen on the membrane surface leads to more negative charge build-up, enhanced hydrophilicity, and polarity [59]. Kim et al. used XPS to calculate the linear fraction and the degree of cross-linking in the PA layer using the following equation:

$$\frac{O}{N}=\;\frac{4n+3m}{2n+3m}$$

where *n* is the linear fraction and *m* is the cross-linked fraction of the PA layer, and *m* + *n* = 1 [105,106]. A similar approach was used to calculate the degree of cross-linking by He et al. [60] and Lee et al. [76].

On the other hand, Yuan et al. reported an increase in the degree of cross-linking of the PA layer after the successful incorporation of covalent organic framework nanosheet (CONs) nanofillers [80]. A similar trend was observed after incorporating polyacrylonitrile (PAN) nanofibers and carbon quantum dots (CQDs) in the PA layer as reported by Shen et al. [107] and Gai et al. [64], respectively.

#### 3.1.3. Energy-Dispersive X-ray Spectroscopy (EDX/EDS)

EDX provides additional information on the membrane elemental composition. EDX penetrates the membrane surface to several micrometers, which is deeper than the XPS penetration depth, thus providing more information on the loading of nanofillers in the PA layer [51,57,108]. In addition, EDX mapping can be used to verify the homogeneity of the nanofiller distribution within the PA layer as specified by Mahmoudi et al. and Li et al. [109,110]. According to the authors, they located EDX peaks at 2.65 and 2.984 cps/eV upon incorporating Ag-GO NPs in their TFN membranes. This indicated the presence of Ag on the membrane surface. In addition to this, they implemented EDX mapping to confirm a uniform distribution of their Ag-GO nanomaterials [109].

### 3.2. Surface and Morphology Analysis

#### 3.2.1. Scanning Electron Microscopy (SEM) and Transmission Electron Microscopy (TEM)

Useful information about the surface structure, PA layer thickness, and morphology of the TFC/TFN membranes can be obtained using SEM and TEM. Cross-sectional images are useful in determining the PA layer thickness and homogeneity of the nanoparticle distribution [60,111]. In general, SEM images of TFN/TFC cross-sections show a dense thin PA layer (~ 100 nm thickness) on the membrane surface, in addition to finger-like pores atop the support layer [85,112]. Furthermore, the surface morphology of TFCs show the typical ridge-valley microstructure due to the formation of the PA layer [110]. Wang et al. reported that CeO_2_ NPs nanofillers were homogenously distributed in the PA thin layer with a change in the membrane surface microstructure. The presence of CeO_2_ nanofillers decreased the thickness of the PA thin layer, which is favorable for improving the water flux through the TFN membrane due to reduced water transfer resistance.

On the other hand, increasing the amount of CeO_2_ NPs nanofillers to 400 mg/L showed an undesirable impact on the PA layer integrity due to NP agglomeration [44]. According to Urper-Bayram et al., the addition of hydrophilic nanofillers, such as Si NPs and TiO_2_ NPs, led to an increase in the membrane porosity and water permeability as evidenced by the SEM images [112]. The same finding was reported for UiO-66-NH_2_ nanofillers by Gohain et al. [74] and for GO/CS (chitosan) core shell nanofillers by Qian et al. [91]. In contrast, the incorporation of hydrophobic CNTs nanofillers in between the support membrane and the PA layer increased the interlayer thickness from 63 to 248 nm and reduced the surface pore size and surface roughness. This was ascribed to the formation of a dense interlayer of CNTs that prevented the diffusion of the amine monomer to the PES support during PA layer formation [84]. Karami et al. reported that TEM images indicated the formation of the PA layer on the surface and inside the pores of the PES support [113]. Additionally, TEM was used by Rashed et al. to image various amounts of CNTs in the PA layer in order to identify CNT aggregation and orientation [71]. While TEM and SEM can be used to directly image nanomaterials in TFNs, other methods such as atomic-force microscopy (AFM) can be employed to probe changes in the structure and morphology of TFNs compared to pristine TFCs. These changes can yield information on the distribution of the embedded nanomaterials.

#### 3.2.2. Atomic-Force Microscopy (AFM)

AFM is used to study the morphological and topographical properties of TFN membranes [114]. As previously stated, TFC membranes show a ridge-valley structure; the addition of different nanofillers increases the surface roughness, which then increases the effective filtration area and improves the water flux [115]. Liu et al. reported that the surface roughness of the membrane increased from 21 nm to 42.7 nm with the addition of 0.1% UiO-66 nanofillers to the PA layer of the TFN membrane [72]. The addition of graphene quantum dot (GQD) nanofillers showed a flatter surface than the TFC membrane, with minor changes in surface roughness from 27.3 to 24.3 nm [116]. Moreover, the precoating of GO on the PSf substrate preceding the formation of the PA layer resulted in the formation of larger nodular structures compared to the TFC. GO preloading on the substrate surface increased the surface hydrophilicity and retained a greater number of amine monomers, allowing for more cross-linking with TMC and improved water flux due to a higher surface roughness [66].

Similar results were reported by Choi et al. for the interlayer coating of polydopamine/graphene oxide (PDA/GO) between a PSf support and the PA layer which increased the surface roughness from 22.5 nm to 37.1 nm [117]. On the contrary, the addition of GO nanofillers to the aqueous phase containing the amine monomer during the PA layer formation led to delayed diffusion of the amine monomer into the organic phase, resulting in the decrease of both surface roughness and PA layer thickness as reported by Zhang et al. [118] and Tian et al. [119]. Similarly, the decrease in surface roughness due to the presence of GO nanofillers was reported by Zhao et al. The authors reported a similar surface roughness between the TFC and TFN membranes containing GO/CNT nanofillers. According to the authors, GO likely led to a smoother surface, while CNTs increased the membrane surface roughness [120].

A smoother membrane surface is associated with anti-fouling and anti-biofouling properties as reported by Lakhotia et al. The addition of CeO_2_ NPs nanofillers decreased the surface roughness from 8.48 nm to 2.02 nm with a simultaneous improvement in the anti-fouling properties of the TFN membranes [48]. Moreover, the incorporation of FeO nanoparticles in the PA layer improved the surface smoothness through the binding of the FeO NPs to the PA layer during the IP process as reported in another investigation by the same group [49]. In addition to using surface roughness, hydrophilicity measurements can be used as an implicit tool for detecting the presence of nanomaterials in the TFN surface.

#### 3.2.3. Contact Angle Measurements

Contact angle measurements are useful in assessing the hydrophilicity of the membrane surface. Lower contact angle values correspond to higher membrane hydrophilicity. To perform contact angle measurements, a drop of deionized water is placed on the membrane surface, and the contact angle is determined by analyzing digital images. To ensure accurate and reliable results, at least four to ten measurements are taken at different positions on the membrane surface [50,121]. Contact angle values below 90° are indicative of a hydrophilic surface [46]. Increasing the hydrophilicity of the membrane is advantageous for enhancing water flux and mitigating the fouling of the membrane [122]. Therefore, contact angle measurements can be used as a tool to detect the effect of hydrophilic nanomaterials, in addition to validating the agglomeration of nanomaterials in the PA layer.

In their investigation, Kotlhao et al. reported that the incorporation of Ag-ZnO nanocomposite nanofillers in the PA layer led to a reduction in the TFC membrane contact angle from 62.8° to 54°, and an increase in water flux from 0.9 (L·m^−^^2^·h^−^^1^·bar^−^^1^) to 1.9 (L·m^−^^2^·h^−^^1^·bar^−^^1^). However, the use of higher amounts of Ag-ZnO nanocomposite nanofillers, exceeding 1.5 wt%, resulted in an increase in the contact angle. This effect was attributed to nanofiller agglomeration and an associated increase in the viscosity of the aqueous phase [85]. Abadikhah et al. reported that the addition of 0.05 wt% GO and 0.2 wt% rGO@TiO_2_@Ag nanocomposite in the PA layer resulted in a reduction of the contact angle to 30° and 21°, respectively, which consequently increased the water flux. This was attributed to the introduction of oxygenated functional groups into the PA layer [123].

According to a recent study by Wang et al., a higher concentration of nanofillers typically results in an increase in the hydrophobicity of the membrane surface, as evidenced by higher contact angles, due to nanofiller agglomeration. On the other hand, the presence of hydrophilic nanofillers and the hydrolysis of unreacted acyl groups in TMC can increase surface hydrophilicity. This results in lower contact angles owing to an increase in the number of hydroxyl and carboxyl functional groups [61]. Al Aani et al. investigated the effect of using carbon nanotubes (CNTs) that were surface-decorated with metal/metal oxide (M/MO) nanoparticles to prepare TFN membranes with improved performance. The additional oxygenated functional groups on the surface of these nanofillers was shown to decrease the contact angle by approximately ~10° [83].

#### 3.2.4. Zeta Potential Measurements

Zeta potential measurements are crucial for understanding the interfacial properties and surface charges of the TFN membranes, which in turn affect the membrane performance, ion selectivity, and fouling [46]. Most membranes have positive zeta potential values at low pH and negative zeta potential values at high pH [124]. Negative surface charges are advantageous for the membrane rejection of divalent and multivalent ions via the Donnan equilibrium [79] and for decreasing biofouling [125]. Typically, TFC membranes have negative zeta potential values above pH 5, which are ascribed to the presence of surface carboxylic acid groups owing to the hydrolysis of unreacted acyl chloride molecules during the IP process [60,126].

Nanomaterials that can impart weak negative surface charges include amine-functionalized nanofillers [73,126], polydopamine-coated silicon nanoparticles (PDA/SiNPs) [88], PDA/TiO_2_ nanoparticles [127], zeolite [61], layered double hydroxide (LDH) [87], and CNTs [84]. On the other hand, more negative zeta potential values for membranes are common for carboxylated and sulfonated functionalized nanofillers [104,126], Ag-MOFs [90], GO [128,129], carbon quantum dots (CQDs) [130], and carboxylated cellulose nanocrystals (C–CNCs) [70]. Hence, zeta potential measurements can be used to validate the presence of certain functional groups embedded in the TFN surface.

#### 3.2.5. Nitrogen Gas Adsorption

Nitrogen gas adsorption can be used for analyzing the pore structure of membranes. The working principle behind the analysis entails the physisorption of nitrogen gas onto the pores to gain insights into the average pore size, average pore surface area, and pore-size distributions. In this respect, the Brunauer–Emmett–Teller analysis is often used to assess the porosity of various nanomaterials in order to characterize their morphology before applying them to the PA layer. For example, Wu et al. used BET to assess the specific surface area of various hydrophobic alkyl-capped silica NPs, and correlated the improvements in dispersibility of the NPs in the PA layer with their BET specific surface area [46]. In another example of nanomaterial analysis, Karki et al. used BET to measure the surface area and pore volume of an amine-rich synthetic talc (NHST) material that was embedded in a polydopamine coating atop a PSf support layer [59]. Rashed et al. used BET analysis to gain insight into the pore-size distribution of their membranes. They used the distribution of the differential pore surface area and differential pore volumes relative to pore width as an indication of membrane pore structure, correlating this to membrane performance [71].

### 3.3. Compositional Analysis

#### 3.3.1. Fourier-Transform Infrared (FTIR)

FTIR is used to identify distinct functional groups present in the membrane. Additionally, FTIR can probe the interactions between the nanofiller and the polymer matrix, and, hence, the integration of the nanofiller in the TFN membranes [120,131]. Typically, the presence of polysulfone (PSf) and polyethersulfone (PES) is evidenced by the bands at 1099 cm^−1^, 1240 cm^−1^, 1150 cm^−1^, and 1330 cm^−1^, corresponding to the stretching vibrations of C-O-C, aromatic ether, O=S=O symmetric, and asymmetric vibrations, respectively [59,86,132]. The presence of an aromatic benzene ring, which characterizes the PES support membrane, is detected at 1485 cm^–1^ and 1578 cm^–1^ as reported in [83]. Upon the addition of nanomaterials, however, the resulting TFN membranes exhibited a reduction in these aromatic bands, which was attributed to the formation of the PA layer as confirmed in a study by Abadikhah et al. [123].

The successful formation of a compact PA layer can be verified by several IR fingerprints. These include the amide C=O stretching vibration at 1660 cm^−1^, the amide C-N stretching vibrations and N-H bending vibration at 1540–1580 cm^−1^, the aromatic amide ring vibrations at 1610 cm^−1^, and the hydrogen-bond-forming amide stretching vibrations at 1490 cm^−1^, as reported in [46,120]. When piperazine is used during the interfacial polymerization (IP), strong bands are observed at 2850 and 2950 cm^−1^, corresponding to the symmetric and asymmetric CH_2_ vibrations of piperazine aliphatic carbons [85]. The incorporation of hydrophilic nanofillers into TFN membranes is associated with an increase in absorption in the range of 3300–3500 cm^−1^, owing to the N-H and O-H stretching vibrations, while absorption of the aromatic stretching vibrations is reduced [133].

#### 3.3.2. Thermal Gravimetric Analysis (TGA)

TGA is used to analyze the thermal stability of the prepared composite membranes by calculating the weight loss on heating the nanocomposite membranes, usually under nitrogen flow conditions. TGA curves of the TFC/TFN membranes involve three steps of weight loss. The first occurs below 180 °C and corresponds to the desorption of water molecules. The second sharp step, at 350–450 °C, is due to the decomposition of the PA layer and loss of functional groups with the release of COx and NOx species. Finally, the destruction of the aromatic skeleton occurs above 600 °C [54]. The improved thermal stability of the PA layers is attributed to the improved crosslinking and the presence of less-unreacted functional groups in the PA layer due to the successful incorporation of the nanofiller, such as functionalized SWCNTs [134], nitrogen-doped graphene oxide quantum dots (N-GOQD) [63], o-hydroxy porous organic polymer (o-POP) [135], MOFs [75,136], TiO_2_/MWCNTs nanocomposites [137], iron oxide (FeO) nanoparticles [49], amine-rich synthetic talc (NHST) nanosheets [59], cerium oxide (CeO_2_) nanoparticles [44], UiO-66-NH_2_ nanoparticles [74], TiO_2_ nanoparticles [43], and palygorskite/Ag nanocomposite [93]. However, the over-addition of the nanofiller can reduce the crosslinking and, consequently, the thermal stability of the prepared TFN membranes in accordance with FTIR and CA findings [63].

#### 3.3.3. Positron Annihilation Spectroscopy (PAS)

Van Goethem et al. used positron annihilation spectroscopy (PAS) to measure the free volume in the PA layer of their prepared membranes. The free volume of the uppermost condensed layer had an influential role in the membrane permeability and performance. The free volume of the PA layer after the successful incorporation of GO was investigated by Lai et al. using PAS. The results showed that the 0.04 g/m^2^ GO-loaded TFN exhibited the highest free volume compared to the TFC membrane and the TFN membranes with lower amounts of GO [66]. However, this technique is limited due to the ridge-valley structure of the PA layer, causing a large spread of data after positron implementation [138].

### 3.4. Mechanical Properties

The mechanical strength and percentage elongation of TFN membranes are crucial for determining the membrane stability and durability after nanofiller incorporation [139]. Lakhotia et al. reported that the optimum loading of CeO_2_ NPs nanofillers increased the tensile strength to 45.2 MPa compared to 13.2 MPa for the bare TFC membrane. This was ascribed to the homogeneous distribution of the nanofiller in the PA layer. In addition, the improvement in the tensile strength of the TFN membranes was accompanied by a 229% increase in the elongation percentage, and showed better cross-linking in the PA layer [48]. However, an increase in the amount of the nanofiller is associated with NP agglomeration, lower mechanical strength, and poor membrane performance [109]. Similar results were observed for FeO NPs [49], Zn/Mg ferrite NPs [52], Ag-GO nanoplates [109], functionalized SWCNTs [134], and for carboxylated cellulose nanocrystals (C–CNCs) [70]. On the other hand, Kotp reported that the incorporation of hydrophilic Al_2_O_3_ NPs decreased the tensile strength of the TFN membranes due to a higher membrane porosity [81].

## 4. Performance of TFN Membranes in Desalination and Water Treatment Applications

TFN membranes are considered a state-of-the-art membrane technology for water treatment. Recent improvements in the TFN membrane performance, antifouling properties, and durability provide new insights and applications for the use of TFN membranes in water treatment applications. As discussed above, the performance of TFN membranes primarily depends on the membrane properties such as porosity, surface roughness, charge, hydrophilicity, and degree of cross-linking. Although water flux and salt rejection are the main criteria for determining the membrane performance, other important factors play a key role, such as antifouling, chlorine resistance, antibacterial, photocatalytic activity, thermal stability, and the removal of dyes and organic pollutants.

Some successful applications of TFN membranes for water treatment are briefly presented below.

### 4.1. Water Flux

Most of the current literature reports that the incorporation of nanoparticles in the polyamide (PA) layer increased the membrane hydrophilicity and decreased the degree of cross-linking in the selective layer. This combined effect improved the water flux through the membrane. Yang et al. attributed the increased water flux, after adding Ag NPs in the aqueous phase during the IP process, to the formation of nanochannels around the embedded Ag NPs. According to their study, the formed nanochannels improved the water flux from 0.93 LMH to 2.50 LMH with enhanced salt rejection. The formation of the 2.5 nm nanochannels around the Ag NPs was confirmed by TEM and was ascribed to the hydrolysis of TMC around the hydrophilic Ag NPs [41]. In a recent study by Seah et al., the incorporation of acrylic-acid-functionalized graphene oxide nanosheets (AA/GO) as nanofillers improved the water flux by 25% compared to the TFC membrane. The increase in water flux was ascribed to its surface hydrophilic nature and the homogenous dispersion of the AA/GO interlayer [69].

### 4.2. Salt Rejection

The membrane salt rejection is governed by the molecular sieving effect, Donnan ion exclusion, and the thickness of the PA layer. The trade-off between water permeability and salt rejection is the main focus for much of the recent research. Lakhotia et al. reported the improvement in the NaCl rejection to more than 90% after the addition of CeO_2_ NPs [48] and FeO NPs [49] in the organic phase during the IP process. This improved salt rejection was accompanied by a slight increase in water permeability and attributed to the enhanced hydrophilicity, roughness, and surface charge of the TFN membranes. The incorporation of CeO_2_ NP-nanofillers decreased the thickness of the PA layer and increased the membrane surface roughness. Consequently, TFN membranes containing CeO_2_ NP-nanofillers could improve the water permeability with minimal effect on the NaCl rejection, as reported by Wang et al. [44]. Other nanofillers have been shown to increase the water permeability with improved salt rejection, such as amine-rich synthetic talc (NHST) nanosheets [59], modified silica (m-silica) NPs [45], MOFs [74,76], and covalent organic framework nanosheets (CONs) [80].

### 4.3. Antifouling

Membrane fouling is a major challenge in membrane technology for water treatment. Chemical and biological fouling reduces the membrane lifetime, durability, and performance with consequent increases in the overall operation cost. Membrane fouling is related to the hydrophobic nature, surface roughness, and negative charge of the TFN membrane [44].

Increased surface hydrophilicity could reduce the adsorption of foulant molecules by forming a stable hydration layer on the membrane surface [140]. In their investigation, Bi et al. used graphene quantum dot (GQD) nanofillers to improve the antifouling properties of the TFN membrane. This antifouling activity was attributed to the weak intermolecular forces and adsorption between the foulants and the membrane surface due to the increased hydrophilicity and reduced surface roughness upon incorporating the nanofillers [141]. Similar results were obtained with other nanofillers, such as MOFs [17,74] and Ag-ZnO nanocomposites [85].

### 4.4. Chlorine Resistance

Chlorine is commonly used as a disinfectant in water treatment applications. However, the rapid deterioration of the PA layer after exposure to strong oxidizing agents such as chlorine created the need to increase the chorine resistance of TFC membranes. The incorporation of nanofillers in the PA layer to form TFN membranes represents a promising way to improve the membrane chlorine resistance by introducing new functional groups onto the membrane surface.

In the work of Shukla et al., the incorporation of a Zn-MOF nanofiller (50 mg/L) in the TFN membranes improved the chlorine resistance of the membrane after a 264 h chlorine exposure. This was attributed to the enhanced hydrophilicity and intermolecular hydrogen bonding between the nanofiller and the PA layer forming a protective layer against a chlorine attack. However, this effect is linked to the homogenous distribution of the nanofiller in the PA matrix, as increasing the amount of the nanofiller resulted in their agglomeration and increased the membrane susceptibility to degradation upon chlorine exposure [136].

The exposure of the PA layer to chlorine was shown to produce N- and ring-chlorination products. These chlorination products caused partial membrane damage and loss of membrane selectivity toward salt rejection. The use of nanofillers with multiple oxygenated functionalities such as cellulose nanofibers (CNF) improved the chlorine resistance of the corresponding TFN membranes due to the formation of more hydrogen bonds with the PA layer [133].

### 4.5. Antibacterial Activity

The antibacterial effect of the incorporated nanofillers is explained by several mechanisms. The formation of reactive oxygen species (ROS) is associated with the UV irradiation of TiO_2_ NPs. As explained by Al Mayyahi, the antibacterial activity against *Escherichia coli* of TFN membranes prepared using TiO_2_ NPs as nanofillers achieved complete sterilization after a 5 h exposure to UV illumination [57]. The interaction of the nanofiller with the bacterial membrane surface was associated with the use of ZnO NPs as nanofillers. In addition, the formation of ROS, which increased with increasing concentrations of ZnO NPs, improved the antibacterial performance against *Escherichia coli* by 91% compared to a blank TFC membrane [58]. TFN membranes impregnated with Ag-MOF [90] and Ag/clay nanocomposites [93] exhibited superior and prolonged antibacterial activity, which was ascribed to the slow release rate of Ag+ ions. Inurria et al. reported that GO-TFN membranes showed less leaching compared to other antimicrobial nanofillers, making this membrane more suitable for biofouling control. The bactericidal effect of GO-TFN membranes was attributed to the physical and chemical interactions between the bacterial cell surface and GO nanosheets on the surface of TFN membranes [142].

### 4.6. Thermal Stability

The thermal stability of TFN membranes is crucial for high-temperature water treatment applications, such as oil recovery plants and the textile industry. Thermally stable membranes can reduce the water treatment process cost and greenhouse gas production [139]. The homogeneous distribution of TiO_2_ NPs in the PA layer improved the thermal resistance of the prepared TFN membrane with improved water flux and antibacterial activity, as reported by Khorshidi et al. [43]. Fathizadeh et al. ascribed the improved thermal stability of the TFN membranes using nitrogen-doped graphene oxide quantum dots (N-GOQD) to the higher cross-linking in the PA layer after the addition of the nanofiller. This was accompanied by a decrease in the amount of the unreacted surface functional group, as evidenced by thermal gravimetric analysis (TGA) measurements [63].

### 4.7. Dye Removal

The use of TFN membranes in organic dye removal from industrial wastewater is attracting significant attention in recent years. Organic dyes are hazardous environmental pollutants due to their toxic and carcinogenic effects. The removal of dyes using TFN membranes is controlled by the molecular sieving effect and Donnan exclusion which depends on the membrane surface charge. He et al. reported the effective use of flower-like MnO_2_ NPs [62] and hollow porous carbon spheres (HPCSs) [68] as nanofillers in the removal of different molecular weight dyes with a removal efficiency of more than 98%. The successful removal of malachite green dye using Zr-MOF nanofillers was attributed to the size exclusion and Donnan ion effect [74].

In their recent work, Tan et al. described the improved performance of TFN membranes using Ag-MOF nanofillers. The TFN membrane exhibited higher flux, antibacterial activity, and significant rejection of organic dyes and divalent ions [90]. The use of carbon nanotubes (CNTs) as interlayers between the polyether sulfone support (PES) and the PA layer improved the membrane rejection of dyes (>99.5%) and divalent ions (>98.3%) with a low rejection of monovalent ions as NaCl (18.8%). The high selectivity of mono/divalent ions and NaCl/dye solution were proven advantageous in the water reuse for textile industries [84].

## 5. Conclusions and Future Outlook

There is an increasing interest in thin-film nanocomposite (TFN) membranes for desalination and water purification applications. While the preparation of membranes with enhanced performance has been the main objective, investigations have also attempted to reach a clearer understanding of the role of nanomaterials in improving membrane performance, and their influence on the physicochemical characteristics of the membranes. To this end, membrane characterization is becoming a crucial component of conducted investigations. As presented in this review, a number of techniques are being used for the structural, surface, and morphological, as well as compositional analysis of the nanocomposite membranes. Attempts are made to relate the membrane characterization results to the effects of incorporating nanomaterials on the one hand and observed changes in membrane performance on the other.

The future prospects of TFN membranes are promising, as they offer a number of advantages over membranes currently used in desalination and water purification applications, namely: improved performance; versatility with the possibility of tuning membrane properties in relation to the different nanofillers used; and durability. However, there are a number of challenges to be addressed. These fall within four primary categories:

- **Nanomaterials used:** While a wide variety of nanomaterials have been employed, as reported in various studies, they represent a small portion of materials which could be used. As more nanomaterials are investigated and used, developing and establishing methodologies for the successful and reproducible incorporation of these different nanofillers within TFN membranes will represent a need of particular significance. More specifically, methodologies for the good dispersion, the controlled orientation, and the possibility of the precise positioning of nanofillers will be needed. Additionally, tailoring the surface properties of nanomaterials for better compatibility with membrane polymer matrices is an area of current research interest that will continue to expand. This will become more relevant with the need to establish an understanding of the role nanofiller interactions with membrane matrices play in membrane performance. Furthermore, enhancing the interactions between nanofillers and polymer matrices would increase the stability and lifespan of TFN membranes, potentially addressing concerns about their environmental impact.
- **Membrane characterization:** While membrane characterization is now more consistently conducted and reported on, a better comprehension of the relation between membrane structure and morphology on the one hand, and membrane performance on the other is very much needed. Furthermore, the impact of the nanofiller presence within membrane matrices on the membrane structure and morphology is still to be consistently investigated and understood. This will necessitate the complementary use of several techniques such as SEM, TEM, FTIR, porometer, and other gas adsorption techniques for pore structure determinations.
- **Long-term performance and impact:** The establishment of TFN membranes as viable alternatives to currently used membranes in desalination and water purification applications is so far limited by a number of factors which will need to be addressed. These include: (i) reproducibility, as producing TFN membranes with consistent performance is still a challenge, and there is still a lack of understanding of the factors that affect reproducibility; (ii) durability—while incorporating nanoparticles in TFN membranes provides increased durability and resistance to fouling, the long-term durability, and, thus, performance, of these membranes is still unknown and needs to be studied; (iii) environmental impact—the leaching of nanomaterials from TFN membranes is a concern for their potential adverse environmental and health implications, and quantifying the leaching rate, toxicity, and exposure risks is very much needed.
- **Scaling-up production and cost:** Scaling up the production of TFN membranes to meet the demands of large-scale water treatment applications would be needed. To this end, the economic viability of TFN membrane production needs to be evaluated for an acceptable cost–benefit balance, as, while TFN membranes are relatively inexpensive to produce compared to other advanced membrane technologies, they are still more expensive than traditional polymeric membranes, which can limit their widespread adoption.

The establishment of TFN membranes in commercial membrane production therefore rests on addressing these challenges in order to successfully develop robust, durable TFN membranes with superior performance which provide a viable alternative for commercial water treatment uses.
